# ‘Being a mother is not child's play’: The capabilities of mothers in a low‐resource setting in South Africa

**DOI:** 10.1111/hex.13689

**Published:** 2023-01-17

**Authors:** Michael Pienaar, Lochner Marais, Mosaathebe Serekoane, Kobus Marais, Jan Cloete, Molefi Lenka, Carla Sharp

**Affiliations:** ^1^ Department of Paediatrics and Child Health University of the Free State Bloemfontein South Africa; ^2^ Centre for Development Support University of the Free State Bloemfontein South Africa; ^3^ Department of Anthropology University of the Free State Bloemfontein South Africa; ^4^ Department of Linguistics and Language Practice University of the Free State Bloemfontein South Africa; ^5^ Department of Psychology University of Houston Houston Texas USA

**Keywords:** 1000 days, capabilities, care for children's health, Children

## Abstract

**Background:**

The importance of a child's first 1000 days has now been widely accepted by the medical fraternity. Yet, we do not know much about caring practices in low‐resource settings.

**Aim:**

This study aimed to investigate the caring capabilities of mothers in a low‐resource setting.

**Method:**

In this study, in‐depth interviews were conducted with 18 mothers with children aged 30 months or younger to better understand the arrangements, means and ends that inform developmental health in a low‐resource setting in South Africa. The study was conducted in a low‐income area, the former black township of Mangaung in Bloemfontein. The mothers were recruited via pamphlets, and two interviews followed. Because of Covid‐19, interviews took place via mobile phones, in Sesotho, the local language in the area. Trained fieldworkers conducted, translated and transcribed the interviews. We used thematic analysis and the capabilities approach as the theoretical framework to analyse the responses from the mothers.

**Findings:**

We used the following organizing themes: pregnancy and ante‐natal care, nutrition, cognitive and physical development, the home environment and access to health care. Although short‐term reactions to pregnancy were often negative, the longer‐term responses showed that the respondents have agency. Most of them could change their nutrition habits, breastfeed and receive adequate nutrition support from the public health system. Most experienced joy when their children reached milestones (cognitive and others), although they became anxious if milestones were not reached. They emphasized children's play and had dreams for their children's futures. Technology was often mentioned as playing a role in their children's development. A large proportion of the respondents had disrupted homes (because of absent or abusive fathers), but some had stable homes. Most of them showed substantial capability to overcome adverse home environments. The public health system helped them deal with their health problems and their children's health problems, although it also created anxiety in many cases. Our data show how they develop their capabilities and overcome obstacles organically in the face of resource limitations. Despite pregnancies being unexpected and unplanned and fathers being absent, the respondents accepted the pregnancy, adjusted their diets and social behaviour, showed agency by attending primary healthcare facilities and ensured that their children received the required vaccinations. Their extended families played an important role in providing care. Despite the sacrifices, the respondents expressed joy and helped their children function by eating, playing, socializing, learning and using their senses.

**Conclusion:**

Our sample of mothers have the agency to adapt to the demands of parenthood and childcare and overcome adversity. Our data support the notion that mothers are held disproportionately and unfairly responsible for achieving the first 1000 days ideals. Despite considerable curtailment of their functionings and capabilities, they nevertheless showed agency to ensure their health and their children's health. A holistic approach should consider these findings in designing policy interventions for children's developmental health.

**Patient and Public Contribution:**

We used paid fieldworkers to interact with the research participants.

## 1 INTRODUCTION

The concept of a child's first 1000 days (from conception to the second birthday) has become prominent in public health. The argument is that if one makes the right ‘investments’ during this period, the child will receive long‐term benefits. The research motivating this argument mainly uses quantitative approaches, with large data sets. The idea of investing in the first 1000 days originates from research showing that the circumstances of the first 3 years of life have a far‐reaching influence on the brain's final anatomical and functional architecture and consequently a person's psycho‐socio‐behavioural functioning.^1^ Events of this early period influence competency and behaviour in adulthood.^2^

Effective interventions during this period may improve children's developmental outcomes and reduce inequality.^3^ However, the biomedical emphasis on the first 1000 days has unfairly pathologized maternal responsibility. By emphasizing biological processes, policies and programmes, this approach tends to neglect the sociopolitical and bio‐political aspects that help or hinder caregivers and families in managing childcare and family life in a way that is meaningful to them. Criticism has been directed at policy interventions that visualize the mother as having no history, that focus on the 1000 days as a target and that aim to enhance the child's prospects while ignoring sociopolitical concerns.^4^

Good nutrition, a stable emotional attachment and the avoidance of toxic stress are primary factors in brain development. Three levels of broader factors are important in a child's development: individual and family (micro), neighbourhood, town and community (meso) and social and political arrangements (macro).^5^ In research on the first 1000 days, a biomedical approach dominates. As a result, this research neglects the issue of caregivers and their contexts, especially in low‐resource settings. In this paper, we argue that qualitative data add valuable perspectives to biomedical data, particularly concerning the demands of parenthood and the responsibilities that the first 1000 days entail. We draw on the capabilities literature to argue for a more complex approach to the mother's role and child development during this period.

Maternal, neonatal and infant deaths are high in low‐resource settings.^6^ Most research focuses on understanding and preventing the problem from a biomedical perspective. Papers and books commonly refer to ‘preventing’ these deaths. Appropriate care is also important for neurocognitive functioning and child development. For example, a systematic review found that play and reading improve neurocognitive functioning in children aged 0–3 years.^7^

However, providing care should go beyond a biomedical approach. Mitchell notes that: ‘Meeting mothers' needs in the care of their babies is important to mothers, family members and professionals, and deserves greater attention’.^8^ A systematic review pointed to the cost‐effectiveness of participatory action and learning to improve maternal and neonatal survival.^9^ Evidence points to the importance of postnatal education programmes^10^ and a dialectical relationship between families and hospital procedures.^8^ Maternal depression is also associated with poor child development outcomes and research has pointed to the importance of lay health workers providing support for women suffering from depression.^11^ Programmes focusing on maternal depression led to better child development outcomes. In a recent study, Garcia et al.^12^ found that, although there was no statistical evidence that fathers' involvement improves child development outcomes, it showed some promise in a low‐resource setting. Research also points to the importance of attending antenatal care visits and breastfeeding for child development.^13^

The biomedical approach focuses largely on the role of care in preventing infant and child mortality. But care can also be seen in broader terms. The African proverb that says ‘it takes a village to raise a child’ embodies this broader approach.^14^ If it takes a village to raise a child, the mother has collective knowledge and support, material and immaterial, that she can harness for childcare. It is our view, this form of capital is a critical part of the immediate eco‐system of the child and essential in the healthy development of the child.

The biomedical focus on mothers' needs is important but lacks an understanding of what mothers do and value and its focus could place undue pressure on mothers to comply with mechanical aspects of caring. Our paper thus uses the capabilities approach to understand what mothers do and value in caring for their children.

The capabilities approach derives from work by Amartya Sen in the 1980s and Martha Nussbaum in the 1990s.^15^, ^16^ Sen^17^ described this approach as ‘an intellectual discipline that gives a central role to the evaluation of a person's achievements and freedoms in terms of his or her actual ability to do the different things a person has reason to value doing or being’. The term ‘Capabilities’ refers to the abilities that allow people to pursue meaningful ends in their lives.^17^, ^18^ They enable people to convert their endowments into meaningful achievements (‘functionings’) and pursue their goals. It thus values meaning and dignity above conventional metrics like income.

The capabilities approach has two central concepts: capabilities and functionings. Capabilities are the freedom and opportunities that people have. Functionings are the doings and beings that people use to achieve their capabilities. For example, travel is a functioning, but freedom to travel is a capability.^18^ People's different abilities to convert resources into functionings are called conversion factors. The resources could be personal (intelligence, reading skills), social (public policies) or environmental (climate, location). The difference between beings and doings is important. ‘Being’ could refer to being a caregiver, while ‘doing’ refers to providing care. In understanding capabilities and functionings, the difference between ends and means is important. For Robeyns, ‘we should always be clear, when valuing something, whether we value it as an end in itself, or as a means to a valuable end’.^18^ Other concepts used in the capabilities approach are agency and structural constraints. Agency is a person's ability to bring about changes, while structural constraints are laws, norms and institutions that inhibit people's capabilities. Research within the capability approach has challenged the biomedical or biostatistical understanding of health,^19^ and particularly as applied to children^20^ and child growth.^21^

In this paper, we argue that qualitative data add valuable perspectives to the biomedical data, particularly concerning the demands of parenthood and the responsibilities that the first 1000 days entail. We draw on the capabilities literature to argue for a more complex approach to the mother's role and child development during this period.

## 2 METHODS

### 2.1 Study area and respondents

Our participants were from the Freedom Square area of the former black township of Mangaung in Bloemfontein, South Africa. Since 1994, Mangaung residents have been able to access property without racial restrictions. The scrapping of the restrictions meant that upper‐middle‐class people moved out of this former black township. According to the 2011 census, the average monthly household income in Mangaung was R5100 (725 USD in 2011).

We recruited respondents by distributing recruitment pamphlets in Freedom Square in 2020, which initially developed as an informal settlement and was upgraded between 1992 and 1994. Household incomes in Freedom Square are even lower than in the wider former township area. Marais and Ntema^22^ recorded an average annual household income here of R22,000 (230 USD in 2008). However, despite the low‐resource setting of our study, mobile technologies and television were commonly available.

All our respondents were mothers of children between 18 and 30 months of age. We did not record their marital status, but their responses give the impression that many did not have a husband or steady partner. Six respondents said that the child's father was making no financial contribution. This meant that they were solely responsible for the child's daily needs and socio‐emotional care. All the respondents depended on the public health system, except for one with private medical aid. However, some respondents occasionally opted to use private medical practitioners. All our participants had access to mobile phones.

### 2.2 Data collection

We trained two fieldworkers who spoke the dominant local language, Sesotho, to conduct the interviews. The fieldworkers conducted the interviews during July and August 2020. Verbal informed consent was obtained from all participants, with the consent documents sent to the participants through Whatsapp (a commonly used messaging service in South Africa). Because of the Covid‐19 lockdown, interviews took place via mobile phones. We set out to do two interviews with each respondent. Respondents received a voucher of R100 per interview for their time. We completed 19 baseline and 18 follow‐up interviews and only used the 18 interviews for which we had both (see Table 1). The interviews were recorded and transcribed and translated by the fieldworkers.

**Table 1:** Profile of the participants

	Still at school?	Gender of child	Number of children	Planned pregnancy	Considered an abortion?	Breastfeeding?	Stopped using alcohol	Giving birth by C‐section	High blood pressure problems	Ref to social grant	Father making a financial contribution
1	No	Male	2	No	No mention	No mention	No mention	C‐section	Yes	No mention	Yes
2	No	Male	3	No	No mention	Yes	No mention	C‐section	Yes	No mention	Yes
3	No	Make	2	No	No mention	Yes	No mention	C‐section	Yes	Yes	No mention
4	No	Male	1	No	Yes	Yes	No mention	No mention	No mention	No mention	No mention
5	No	Female	3	Yes	No mention	Tried, had problems	No mention	No mention	Yes	No mention	Yes
6	No	Female	3	No	No mention	Yes	Yes	No mention	No	Yes	No
7	Yes	Female	2	No	Yes	No mention	Yes	No mention	No mention	Yes	No
8	No	Not available	Not available	No	No mention	First 20 days, then got sick	No mention	C‐section	Yes	Yes	No
9	No	Not available	Not available	No	No mention	Yes	No mention	C‐section	No mention	No mention	Yes
10	No	Not available	Not available	No	No mention	Yes	No mention	No mention	No mention	No mention	No
11	No	Male	2	Yes	No mention	Yes, but stopped because the child was not gaining wait	Yes	C‐section	No mention	No mention	Yes
12	No	Male	Not available	No mention	No mention	Yes	Yes	No mention	No mention	No mention	Yes
13	No	Male	Not available	No	No mention	No mention	No mention	No mention	No mention	No mention	No
14	No	Not available	Not available	No mention	No mention	Yes	No mention	No mention	No mention	Yes	No‐mention
15	Yes	Not available	Not available	No	No mention	Yes	No mention	No mention	No mention	No mention	Yes
16	No	Not available	1	No	No mention	Yes	No mention	No mention	No mention	No mention	Yes
17	No	Male	1	No	No mention	Yes	No mention	No mention	No mention	No mention	Yes
18	No	Male	4	No mention	No mention	Yes	No mention	C‐section	Yes	No mention	No mention

### 2.3 The interview framework

We asked one question during the first interview: ‘Tell us the story of raising this child’. We probed for the mothers' wishes for their child, the extent to which these wishes were realized and the boons and difficulties of raising a child. The interviews were conducted to elicit the mother's story of her pregnancy, her child's birth and the first 2 years of her child's life, and fieldworkers were trained to probe selectively. During the second interview (about 2 weeks later), the interviewers clarified any unclear information from the first interview and asked six additional questions drawn from our reading of the first round of transcribed interviews:

(1) How often did the child get sick, and what did you do when the child was sick?
(2) What was the most challenging aspect of raising this child?
(3) What was your main achievement in raising this child?
(4) What did you do to help the child to develop physically?
(5) What did you do to promote the child's brain development?
(6) Is someone else looking after the child regularly? Why, who and why do you trust this person?

### 2.4 Data analysis

We used thematic analysis, starting with themes from the literature of the first 1000 days and amending the themes on the basis of the mothers' stories. The first two authors conducted coding independently and then discussed it to settle on a common set of themes. A preliminary coding framework was developed from the literature. The transcripts were dissected using the preliminary framework. Abstract themes were first identified, followed by elegant themes. The themes were then arranged into a network to distinguish basic and organizing themes.^23^

## 3 FINDINGS

We discuss the experience of mothers within the five essentials of the first 1000 days as our organizing themes: pregnancy and ante‐natal care, nutrition, cognitive and physical development, home environment and access to health care.

### 3.1 Pregnancy and ante‐natal care

Ante‐natal care and maternal health are key policy concerns in South Africa, where the maternal death rate remains high.^24^ We identified two subthemes: short‐term reaction to falling pregnant and longer‐term responses. Of our 18 respondents, 13 spoke about aspects of falling pregnant, and 10 of these had had unplanned pregnancies. They said things like ‘I was angry’, ‘I was not happy’, ‘I was shocked’, ‘I did not want the child’, ‘It wasted my time’, ‘It took time to pull myself together’, ‘I was surprised’ and ‘I was afraid’. A few respondents had not immediately realized that they were pregnant. One of them had visited the clinic for hypertension and only then discovered that she was 6 months pregnant. Two respondents had considered abortion but decided against it on religious grounds. Two said that pregnancy had affected their education. However, three respondents expressed their excitement at falling pregnant.

Despite about half of the respondents falling pregnant unintentionally, most of them had the capability to accept the pregnancy eventually, seek medical care and bond with the child. Respondents used words like ‘excitement’ and ‘a joyful moment’. One of them described how she got used to being pregnant, felt more at ease, started getting excited about the baby and was very happy when the baby was born.

### 3.2 Nutrition

We identified three subthemes regarding nutrition: behaviour change during pregnancy, breastfeeding and the role of the public health system in monitoring children's diets. Many of our respondents said that falling pregnant forced them to change their behaviour, for example, by stopping drinking alcohol and starting to eat healthily ‘for the sake of the child’. Concerning alcohol, one respondent said:

I could stop drinking alcohol so I could raise my child properly, with respect. Because when you're drunk, you get out of control, you know? You don't even know how to speak correctly, and even the child won't know you because you are drunk. So that is something I wanted to change.

This respondent did not relate the use of alcohol to foetal alcohol syndrome. Instead, her motivation was to become a responsible parent.

Breastfeeding was common because staff at the public clinic emphasized the importance of breastfeeding. Respondents were able to explain the nutritional value of breastfeeding because they had visited a public clinic. However, going back to work was a problem for continued breastfeeding, as one explained:

When he was still with me before I went back to work, he was breastfeeding and did not want a bottle. He never got used to it—he refused it. And when the child drinks milk from a cup, it is different from when he is breastfeed. So, he lost weight a little bit.

The clinic staff weighed the children and recorded weight gains or losses. Respondents demonstrated agency in trying hard to comply with advice from the clinic despite practical realities and the lack of accommodation for breastfeeding mothers in the workplace. Knowing that the nurse would weigh the baby made some respondents anxious. They felt that the nurses scolded them when there was little increase in the child's weight. Finally, several respondents talked about the difficulty of affording food and having to go hungry but ensuring that the child had food.

### 3.3 Cognitive and physical development

We identified the following subthemes: reactions to reaching or not reaching milestones, an understanding of the importance of play, the role of the public health system, dreams for their children and the role of technology. Most respondents had an idea of children's expected development milestones, such as sitting up at about 6 months, walking at about 1 year, recognizing their names and saying words. They enjoyed seeing their child reach these milestones, expressing delight if the child was crawling and standing very young and excitement at seeing them grow and talk. These expressions of joy stood in contrast to their descriptions of surprise and fear when they found out that they were pregnant. However, external organizations can overemphasize these milestones, causing anxiety. Some of our respondents had worried that not achieving the goals according to the guidelines meant that something was wrong.

Most respondents talked about their children's play (identified as a capability by Nussbaum).^15^ Some of them related play to social development and learning, as the following quotes seem to show:

I love playing with kids, spend some of my time playing with them, and you know that a child grows very well when playing with other kids.

Sometimes I leave him to play free but making sure there is no danger. When he wants to touch things, I let him be so that he can feel different things.

However, none of the respondents mentioned that play contributes to cognitive development. None of them said that they had been told this by the clinics or doctors. Safety was a primary consideration. One respondent mentioned locking house doors to keep the child safe.

Opinions were divided about how much the clinic staff had helped them understand and foster physical and cognitive development. Some felt that the staff had been extremely helpful. One respondent said:

The clinic showed us how to speak to the child normally and said we need not use baby language. He will understand. I think that helped. Because now when I talk to him he responds, even when he responds with his eyes. The simple thing is making a conversation with him because language development is important. When you bath him, talk to him. Tell him: ‘I'm bathing you, clothing you or putting on your shoes’—such simple things.

This quote emphasizes the importance of conversations with the child and confirms that this message originated from the primary health system. This understanding that normal daily activities contribute to physical and cognitive development and connect the child to people in the household is important in a low‐resource setting.

Some respondents believed that crèches and schools were crucial for physical and cognitive development. One said that her child was not learning anything when he was at home and that at the crèche, he would ‘be able to learn something new every day’. Another explained her decision as follows:

I think the importance of taking a child to crèche is so that he can start writing at a young age. So he can know the right way to hold a pen—not necessarily writing, but how to hold a pen. Also, knowing how to play with building blocks so that he may know how to build things with his hands.

Despite the value that mothers saw in early childhood development (ECD) centres, some expressed concerns about the quality of care. However, many respondents were worried about the cost of sending the child to a good crèche or school.

Many respondents had dreams for their children. The following quote summarizes the feeling of most of them:

My dreams about my children are to see them achieving more things than I have achieved myself. I want my children to study further. I want them to be respectful to people because one can be educated and all, but not have a respectful nature, and they are just nothing without respect.

Respondents also mentioned the advantage, and possible disadvantage, of technology. We heard comments such as ‘he is fighting for the phone’, ‘he knows everyone's phone in the house’, ‘he loves watching cartoons’ and he ‘repeats the songs from the TV’. Although overexposure could be negative, these endowments do have the advantage of connecting the children to the outside world.

However, beyond the discussions about achieving good physical and cognitive development, perhaps the best summing up was from the respondent who described her achievement as just ‘seeing him being okay, seeing him around and him being here with me’.

### 3.4 Home environment

We identified three subthemes: disrupters, stabilizing factors and the mother's agency to overcome adverse home environments. The two main disrupters were neglect by the children's fathers and financial distress. At least half of the respondents had difficult relationships with partners who did not acknowledge the child, did not contribute financially to raising the child or abused alcohol. One respondent said: ‘My wish was that the love that I give the baby, I wished that the father would give it as well’.

Financial concerns included paying someone to look after the child and buying formula milk, diapers and creams to avoid rashes. The distress caused by money worries was evident in one respondent's words:

When my children need something, I can't give them that thing. I can't give it to them even if I want to. Things like that make me wish that I didn't have children. I think that's the most painful thing.

Several respondents mentioned financial sacrifices like walking rather than using a taxi, and not buying formula milk and baby food but putting the child on homemade food early. However, despite this lack of support from fathers and financial difficulties, our respondents showed agency. We heard many comments like this about the fathers: ‘I don't even care about him’, ‘I have pride’, ‘I'll raise the baby for the father’, ‘I am going to carry my problems’ and ‘I was trying to do everything myself’.

The respondents mentioned several stabilizing factors: a good relationship with their partner, help from the extended family, a trustworthy caregiver and the South African child support grant.

A positive relationship with the child's father was seen as especially important during pregnancy. One respondent said that she ‘had someone who was supporting me throughout my pregnancy, so I ended up enjoying being pregnant’, and it helped that ‘at least his father listens to me when I talk to him’. Another said that when she came back home with the baby, ‘the baby's father was the one who looked after us, just the two of us’.

Respondents mentioned their mothers and grandmothers in particular as important sources of care and financial support for the children. One respondent said:

I receive support from both sides, especially from his grandmother. She loves him. She is very protective of the home. When I tell her that the child is sick, she comes with everything.

Respondents said that it was not easy to find the right person to care for the child when they were at work. But most were satisfied, saying that they trusted this person, that the child was clean, the person is taking good care of the child, the child is growing well and even that they received good advice from the caregiver.

A few respondents mentioned the stabilizing role of the child support grant that the South African government provides. They thought that the amount was too small (26 USD at the time of the study), but were nevertheless most thankful for the contribution. Some used it to buy formula milk, diapers or food or to pay for babysitting.

Most respondents mentioned that attachment developed between them and their children. They said things like ‘we are very close’, and described children following them, sharing telephone discussions and not wanting to leave them. One of the mothers stressed the importance of mother–child bonding, saying that if ‘it is not okay’, the child will be mentally harmed.

In a low‐resource setting, exercising agency often involves making sacrifices. They spoke about not getting enough sleep, being tired and not having enough time. They spoke of household chores, headaches and nerve problems, and referred to the child as a ‘load to carry’. They spoke of crying and feeling overwhelmed. On the social side, they spoke of a diminished social life, fewer friends (and dating), routine changes, reduced alcohol intake, missing out on breakfast or an afternoon nap, limited freedom and being controlled by the child. One mother said, while laughing: ‘Being a mother is not child's play [Sesotho, *Ho ba mme ha se papadi*—literally, “To be a mother is not a game”]’. She explained as follows: ‘Because, everything you do, you have to think about the child’. Another referred to ‘baby stress’.

### 3.5 Access to health care

In our analysis of the interview transcripts, we identified three main subthemes of health care: health problems during pregnancy, child health problems and the role of the health system, public or private, in managing the problems.

Maternal death at birth is abnormally high in South Africa, at 100 deaths per 100,000 live births.^25^ Respondents reported several health concerns. Six mentioned gestational hypertension. Seven had given birth by Caesarean section. Other problems during pregnancy that they mentioned were excessive vaginal discharge, heartburn, sleeplessness, excessive sleeping, pressure on a hip joint, dizziness, feeling weak, back pain, headaches, abdominal pain, vomiting and feeling ill. One mentioned a benign tumour.

There were 18 references to stress during pregnancy. Various reasons cited for the stress were the pregnancy being unplanned, not accepting the situation, work‐related difficulties such as travelling, worry about the financial situation (how to provide for the baby after birth) and lack of support from the child's father.

Some mentioned feeling depressed following delivery. One respondent said: ‘After having the baby, I had convinced myself that there was nothing else I could do. I had given up on life’.

Access to adequate health care and skilled providers is key to maternal and child health. Respondents reported mixed experiences in attending ante‐natal clinics. One respondent expressed her diligence as follows:

Once the doctor confirmed that I was pregnant, I started attending ante‐natal classes. I went to the clinic as early as possible so that if something was wrong with the baby—they can see it earlier, you see? I didn't miss my appointments. I made sure to take the pills the way they told me to. I even changed my diet, a little.

The respondents were generally thankful for receiving appropriate services from the public health service and most said that once they received confirmation of pregnancy, they attended according to their programme. Two said that they appreciated the text messages reminding them about their appointments.

The respondents were generally aware of their children's needs. They mentioned respiratory problems, including pneumonia, and urinary and teething problems. Some reported only minimal child health problems. All the respondents emphasized that they wanted their children to grow up healthy. One mother said that to achieve this, ‘I will have to look after him properly’.

The respondents described many worries. One said, ‘When he is still a baby and just cries the whole day, you worry’. Another said, ‘He would get sick, probably, two to three times in a month’. A child's illness became a stress factor for the mother. One respondent said that this stressed her because ‘I would feel like I should have been the one who was sick, not him’. Respondents noted that the frequency of illnesses decreases as the child grows older: ‘After he turns a year old, then those problems become less’. Three respondents reported serious health problems that required the child to be hospitalized, saying that ‘it was scary’ and ‘I was in pain’.

Despite these concerns about the child, we heard many remarks indicative of the respondents' resourcefulness, in other words, agency, in managing difficult circumstances. One respondent said: ‘If the child doesn't want to eat, you shouldn't give up. You have to be patient with your child’. Another said that since the child was a baby, she had ‘tried, by all means, to ensure that he grows up healthy’.

Respondents mostly went to a primary healthcare clinic when the child was sick or, for those who could afford it, a private medical practitioner. Respondents attended the postnatal clinic for the child's EPI vaccines and for advice, as one explained:

He had to have vaccinations according to the book ‘Road to Health’, from three days to six weeks—injections and those drops. The second thing was tracking his growth, weight, height and seeing if he had reached milestones and was not delayed or being held back. They would ask if he could respond to my gestures, and smile and laugh. If you don't attend those sessions, you won't know when something is wrong with your child.

However, despite the clinics, doctors and Google, misconceptions remained about what causes health problems. One mother blamed herself, and reported some astonishing misinformation that she had received from a clinic:

When this child was sick, I would worry a lot because his flu was too much. He had a problem with his chest and lost his breath. We always had to spend money to get to the doctor. I think he had flu because I liked cold stuff. I used to eat a lot of cold stuff, and it affects the baby, so when the baby is born, they are born having flu.

Affording private medical care was a problem for many mothers. Considering some of the resource‐related problems explained earlier, this is understandable. Several respondents complained about the cost of health care and medicines.

One respondent spoke about getting the necessary support ‘at the right time’, and said that going to the clinic made her aware of her responsibility. However, their views on service quality at the postnatal clinics were mixed.

Some respondents related instances of inadequate service from the public health facilities. One said that after not receiving adequate care from the public health system, one of the private hospitals helped her. Another said: ‘When I take my child to a doctor, they check him from head to toe. They make sure that they don't miss anything’. We heard complaints about poor treatment, such as: ‘They didn't say much. They said I should bring the baby for his injections. If he doesn't get his injection, he will fall ill’. One respondent complained about the clinic staff not believing her when she said that her child could not urinate. Some respondents were scared of the nurses: ‘But if you dare miss your clinic date—yoh! They spit fire’.

## 4 DISCUSSION AND CONCLUSION

In this discussion, we analyse our findings within the capabilities approach and against the literature on the first 1000 days and caring (see Table 2).

**Table 2:** Summary of the main findings

	Functionings (achievements)	Conversion factors	Capabilities
Related to the mother during pregnancy			
Doings	Falling pregnant Attending ante‐natal clinics Getting a diagnosis for underlying illness Getting advice from other people.	*Personal* Personal reaction to being pregnant Adjusting behaviour	Being healthy as a metacapability Enjoying pregnancy Competing capabilities—opportunities for education or employment, social life, etc.
	Getting inputs/help from life partners or family (or not) Adjusting their diets Adjusting their social behaviour	*Social* Following advice from ante‐natal clinics	
	Considering abortion Giving birth safely	*Environmental* Near primary healthcare facilities/hospital	
Beings	Being healthy Accepting being pregnant and enjoying it		
Related to the mother–child relationship			
Doings	Attending primary healthcare facilities for children Obtaining and using information from primary healthcare facilities	*Personal* Decision to care for the child	Child is healthy Child grows/develops/reaches milestones Child shows cognitive development Mothers talk about long‐term capabilities for the children
	Facilitating child play Providing the child with nutrition Facilitating learning Breastfeeding	*Social* Following advice from postnatal clinics	
	Adjusting to work and taking care of the child at the same time Speaking to the child Making eye contact with the child Providing care despite father's absence Accessing social support for raising the child Providing education opportunities Returning to work	*Environmental* Near primary healthcare facilities/hospital	
Beings	Child is well‐nourished Bonding with the child Enjoying having the child Making sacrifices		
Related to the child			
Doings	Play Opportunities to learn Eating	*Personal* Access to food	Child is healthy Child grows/develops/reaches milestones Child shows good cognitive development
	Socializing Growth Using their senses	*Social* Social, learning and play opportunities	
Beings	Well‐nourished Having social interaction		
	Bonding with the child Dreaming about the child's future	*Environmental* Near primary healthcare facilities/hospital	
Examples of agency	Changing eating and drinking behaviour when pregnancy was confirmed Accepting being pregnant and enjoying it and finding joy in the child's growth Finding an appropriate caregiver when returning to work Sacrificing own needs Objective to the rudeness of clinic staff		
Structural constraints (sociopolitical arrangements that inhibit capabilities)	Impact of pregnancy on education Absent or unsupportive fathers Lack of resources Unsupportive workplaces Incorrect information from clinics		

The health outcomes recommended in the first 1000 days literature require certain functionings by the mothers. Examples of these include going to a clinic or doctor during pregnancy. Most of our respondents were late in confirming their pregnancy and half of them had unplanned pregnancies. However, once they and their children were part of the primary healthcare system, they remained in the system. Most respondents spoke about adjusting their diets and social behaviour after falling pregnant. There was a concerted effort to stay healthy during pregnancy. Despite pregnancies being unplanned, all the respondents eventually accepted being pregnant and enjoyed it (a capability identified by Nussbaum).^15^ This behaviour confirms the literature that shows the importance of attending antenatal healthcare facilities^13^ and confirms Nussbaum's^26^ capabilities related to emotions. It is possible to link postnatal care and mothers and such a link would benefit the children.

We noted important functionings and capabilities related to the relationship between the mother and the child. Doings include attending primary healthcare facilities with the child and ensuring that the child was vaccinated. Most respondents realized the importance of providing good nutrition, allowing their children to play and facilitating learning by creating educational opportunities. The mother's functioning supports child development and confirms the literature on the importance of attending antenatal clinics^13^ and the role of play in the cognitive development of the child.^7^ Some mentioned the value of talking to the child and making eye contact—both important for neural development. Despite absent fathers, respondents said that they were able to care for their children on their own and with the help of extended families, confirming the important roles of the extended family noted in the literature. The literature shows that although the father's involvement is important, most mothers will be able to raise children adequately. Our evidence shows that mothers are doing this and that they find support from the grandmother and extended families and the village.^12^ Most of the responses suggest that the child was well nourished and the mother had bonded with the child. Despite making sacrifices, most respondents said that they enjoyed having the child. All of this gave them the capability to promote their child's growth and cognitive development. It enabled them to dream about the child's future. In the respondents' descriptions of the children's behaviour, we noted several functionings: eating, playing, socializing, learning and using their senses. Most of these are simple requirements for the cognitive and child development indicators identified by research. These functionings lead to growth, health and cognitive development capabilities. Despite several structural constraints, we found many examples of the respondents exercising agency.

We think that there are three specific values attached to the use of a qualitative approach. First, the respondents' stories brought to light their ability to change their behaviour. Biomedical literature often emphasizes interventions to change behaviour and ignores the agency that people have. For example, the first 1000 days literature stresses the importance of ante‐natal care, maternal health (in a primarily biomedical sense) and a stable home environment. This can become a mechanical way of thinking about raising children. Our approach allowed us to understand how mothers can deal positively with difficult circumstances during the first 1000 days.

Second, linked to the above, the first 1000 days literature's expectations could give mothers a sense of failure, a common problem identified in the literature.^4^ Although our respondents did receive some very good advice from the public health system, some of them feared going to the clinic to be reprimanded by the staff. The responses show how focusing on some metrics could deter mothers from going to the clinic. Although the literature points to the important role of antenatal clinics,^13^ our evidence shows that the nature of the support from these clinics is crucial in keeping women in the system.

Third, the 1000 days literature tends to overemphasize specific health issues and does not always take a holistic approach. For example, respondents referred to their children achieving the capability of play. Yet, none of them related play to cognitive development. From their responses, we could not detect that the public health facilities emphasized play. One mother reported being advised to talk to her child regularly. We would like to see more of these less mechanistic approaches supported by the health system.

Although we do not intend to generalize from our small sample, we acknowledge that our recruitment method may have caused some bias. The voucher that we provided may mean that we attracted a disproportionate number of respondents who were very poor. Our sample may also be disproportionately made up of women who were managing motherhood well, in other words displayed agency, as those who were struggling might not have had the energy to participate in the study, or been eager to reveal their failure.

In conclusion, we would argue that our respondents' evidence supports the notion that mothers are held disproportionately and unfairly responsible for achieving the first 1000 days ideals. Many issues that make it difficult for mothers to enhance their children's developmental health are not internal or personal. Rather, they are the result of negative social arrangements such as the absence of supportive fathers, work environments that do not support mothers returning to work and inequalities (racial, gender and otherwise) in the quality of services available to mothers. The constraints within a low‐resource setting place unfair pressure on mothers to provide for their children's daily needs and facilitate socio‐emotional stability, physical growth and cognitive development. They have to manage with limited means and are constrained in their ability to convert these means into the desired end: their children's developmental health. The 18 mothers in our sample experienced a disproportionate curtailment of their functionings and capabilities. A holistic approach should take this into account in designing policy interventions for children's developmental health.

## AUTHOR CONTRIBUTIONS

**Michael Pienaar**: Conceptualization; formal analysis; writing – original draft. **Lochner Marais**: Conceptualization; formal analysis; writing – original draft. **Mosaathebe Serekoane**: Conceptualization; data curation; methodology; writing – review and editing. **Kobus Marais**: Conceptualization; writing – review and editing. **Jan Cloete**: Conceptualization; data curation; methodology; writing – review and editing. **Molefi Lenka**: Conceptualization; data curation. **Carla Sharp**: Conceptualization; writing – review and editing.

## CONFLICT OF INTEREST

The authors declare no conflict of interest.

## ETHICS STATEMENT

The research has ethics approval from the Faculty of Health Science Ethics Committee at the University of the Free State. Participants provided consent.

## Data Availability

The data that support the findings of this study are available from the corresponding author upon reasonable request.
